# Red Pepper Powder Enhances Antioxidant and Immune Functions in the Sea Urchin *Strongylocentrotus intermedius*: Potential as a Functional Feed in Aquaculture

**DOI:** 10.3390/antiox14101173

**Published:** 2025-09-26

**Authors:** Jiadong Guo, Yuntian Zhang, Yi Chen, Yupeng Zhang, Rongwei Zhang, Yuzhe Han, Xiaoran Zhao, Tongjun Ren

**Affiliations:** 1 College of Fisheries and Life Science, Dalian Ocean University, 52 Heishijiao Street, Shahekou District, Dalian 116023, China; jiadongguo28@gmail.com (J.G.); zyt13803202823@163.com (Y.Z.); 17685270905@163.com (Y.C.); zyupeng317@gmail.com (Y.Z.); zhangrongwei1017@163.com (R.Z.); hyz@dlou.edu.cn (Y.H.); zhaoxiaoran@dlou.edu.cn (X.Z.); 2 Dalian Key Laboratory of Breeding, Reproduction and Aquaculture of Crustaceans, Shahekou District, Dalian 116023, China

**Keywords:** *Strongylocentrotus intermedius*, red pepper powder, growth performance, antioxidant function, immune response, stress mitigation

## Abstract

Driven by the concept of sustainable aquaculture, natural feed additives with growth-promoting, antioxidant, and immune-enhancing properties have become a key research focus. This study assessed the effects of dietary red pepper powder (*Capsicum annuum*) supplementation at 0%, 0.5%, 1.0%, and 2.0% over 50 days on the growth, digestive function, immune and antioxidant capacities, intestinal microbiota, and gene expression in *Strongylocentrotus intermedius* (*S. intermedius*). The results indicated that red pepper powder significantly promoted growth and decreased the feed conversion ratio (FCR) (*p* < 0.05), with the 1.0% group showing the highest growth rate. Additionally, supplementation improved gonadal coloration and increased crude protein and lipid contents in the gonads, particularly in the 1.0% and 2.0% groups (*p* < 0.05). Supplementation with 1.0% and 2.0% red pepper powder enhanced digestive, immune, and antioxidant enzyme activities, while reducing malondialdehyde (MDA) levels, indicating lower lipid peroxidation. α-diversity analysis revealed the highest ACE, Chao, and Shannon indices and the lowest Simpson index in the 1.0% group, indicating greater microbial diversity. Community analysis revealed that in the red pepper powder treatment groups, beneficial bacteria, such as *Firmicutes* and *Unclassified_f__Rhodobacteraceae*, increased in relative abundance, while potential pathogens like *Arcobacter*, and *Epsilonbacteraeota* were less abundant. Red pepper powder supplementation upregulated key immune- and antioxidant-related genes while downregulating pro-inflammatory and stress-associated genes. Overall, optimal dietary supplementation of red pepper powder, particularly at 1.0%, enhanced antioxidant and immune functions, optimized intestinal microbiota, mitigated oxidative stress, and consequently promoted growth, improved gonadal quality, and strengthened overall health in *S. intermedius*.

## 1. Introduction

The intermediate sea urchin (*Strongylocentrotus intermedius*) is widely distributed along the subarctic coastal waters of the North Pacific and is an echinoderm of significant ecological and economic value. The gonads are nutritionally rich, containing abundant amino acids, polysaccharides, lipids, vitamins, and trace minerals, thereby representing a valuable food resource [1]. In recent decades, overfishing and climate change have led to a sharp decline in wild populations, threatening the sustainable use of sea urchin resources, disrupting local marine ecosystems, and hindering the development of related industries. To meet the growing market demand, the scale of intermediate sea urchin farming has rapidly expanded, with intensive farming systems becoming the dominant production model [2,3]. Although intensive aquaculture systems significantly increase yield per unit area by optimizing resource utilization, sea urchins in high-density environments endure continuous biological and abiotic stress. Such stressful conditions may disrupt their internal homeostasis, promoting the excessive production of reactive oxygen species (ROS), which triggers oxidative stress responses [4]. There is a complex interaction between oxidative stress and inflammation, with the NF-κB and MAPK signaling pathways facilitating the release of pro-inflammatory factors, leading to significant damage to macromolecules (such as proteins, lipids, and DNA) and weakening immune defenses [5]. The persistent activation of oxidative stress and inflammatory responses poses a substantial threat to the health of sea urchins, making the search for effective antioxidant treatments a key aspect of sustainable aquaculture.

In this context, natural medicinal plants, with their significant biological activities such as antioxidant, antibacterial, and anti-inflammatory properties, have become a major focus of functional dietary supplement research [6,7,8]. Whether used as whole plants, specific plant tissues, or bioactive extracts, these plant compounds have shown considerable potential in enhancing the antioxidant capacity and immune response of aquatic animals, while offering advantages in environmental safety and economic feasibility [9,10,11,12]. Among various natural medicinal plants, the Solanaceae species, particularly *Capsicum annuum* (red pepper), is especially rich in a variety of bioactive compounds, including vitamins A, C, E, β-carotene, capsaicin, flavonoids, and tannins [13,14]. These components exhibit strong antioxidant and immune-modulatory effects, capable of scavenging reactive oxygen species (ROS) generated during metabolism, mitigating oxidative stress, and thereby maintaining cellular function and overall health [15,16,17]. Carotenoids, as precursors to vitamins, play a crucial physiological role, particularly in promoting growth and maintaining health, while their antioxidant properties help protect macromolecules in cells from oxidative damage [18,19]. Previous studies have shown that red pepper not only has significant anti-inflammatory and antioxidant effects but also significantly promotes poultry growth and optimizes digestive efficiency, making it a promising alternative to antibiotics [20]. Additionally, research has found that the addition of *Capsicum annuum* to the feed of rainbow trout (*Oncorhynchus mykiss*) improves growth performance, enhances intestinal microbiota diversity, and boosts immune function [21]. Although numerous studies have focused on the benefits of dietary red pepper, most have been centered on fish and poultry [22,23], with molecular-level studies on echinoderms, particularly *S. intermedius*, still lacking.

This study aims to investigate the effects of dietary red pepper powder on the growth performance, gonad quality, antioxidant and immune responses, intestinal microbiota composition, and the expression of immune (*TLR*, *LYZ*), antioxidant (*GPX*, *GST*), inflammatory (*COX-2*, *TNF-α*), and stress-related (*HSP70*, *HSF1*) genes in *S. intermedius*. The study also seeks to uncover the potential mechanisms by which medicinal plants enhance antioxidant defense systems and immune capacity, reduce oxidative damage, thereby alleviating oxidative stress and promoting healthy aquaculture.

## 2. Materials and Methods

### 2.1. Experimental Diets and Analysis

The red pepper applied in this study was procured from Fuxitang Pharmaceutical Co., Ltd. (Meishan City, China), dried, ground, and sieved through a 120-mesh screen to obtain feed-grade powder. Three experimental diets were formulated: LL group, LM group, and LH group, containing 0.5%, 1.0%, and 2.0% red pepper powder, respectively, based on a basal control group feed (LC group, 0%). The formulation composition and proximate nutrient composition of the diets are presented in Table 1, and all diets were nutritionally balanced to support healthy growth in sea urchins [24,25,26]. All dried components were finely milled, passed through an 80-mesh sieve, and thoroughly mixed according to the specified ratios. The mixture was then combined with water at a dry matter to water ratio of 7:3 to form a paste, which was pelletized into sheet-form feed using a tableting machine (Model MT-40-I, Xianghe Dough Processing Machinery Co., Ltd., Xingtai, China). The resulting feed was air-dried at 55 °C, cooled naturally to ambient conditions, individually packaged, and preserved at −20 °C until use.

The proximate composition of the experimental diets (crude protein, lipid, ash, and moisture) was determined following recognized procedures established by the AOAC [27]. Moisture content was measured by drying the samples at 105 °C for 24 h in a laboratory oven (101-3BS, Shangcheng Instruments, Shanghai, China). Ash content was determined by incinerating the samples at 550 °C for 6 h in a muffle furnace (MFLX325-14, Muffle Furnace Tech, Shanghai, China). Crude protein content was analyzed with an automatic nitrogen analyzer (KDN-1000, Xinjia Instruments, Shanghai, China), with nitrogen values converted to protein using a factor of N × 6.25. Crude lipid content was quantified through the application of a Soxhlet extractor (Model QWSZF-06A, Qiwei Instruments, Shishi City, China). Gross energy was quantified with an adiabatic bomb calorimeter (C2000, IKA Werke, Staufen, Germany).

### 2.2. Experimental Design

**Figure 1:**
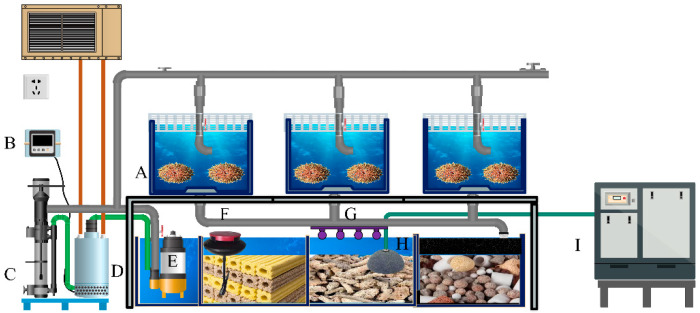
Schematic diagram of the recirculating aquaculture system. (**A**) Sea urchin tank; (**B**) Temperature controller; (**C**) Protein skimmer; (**D**) Chiller; (**E**) Water pump; (**F**) Online fluorescence dissolved oxygen meter; (**G**) Ultraviolet Sterilization Lamp; (**H**) Air diffuser disc; (**I**) Oxygen Pump.

The control and treatment groups were reared in independent recirculating aquaculture systems (RAS), with identical conditions maintained in each system to ensure comparability between groups (Figure 1). Water quality was stabilized through mechanical and biological filtration combined with ultraviolet disinfection, ensuring physicochemical consistency and biosecurity [28]. Each tank was equipped with separate inlet and outlet systems to guarantee water independence and improve the reproducibility of the results.

The *S. intermedius* used in this study were obtained from Dalian Yinhaima Products Co., Ltd. Before initiating the experiment, the *S. intermedius* were acclimated in holding tanks for two weeks to minimize the impact of environmental changes on their physiological condition. Randomly selected individuals were examined under a light microscope for external surfaces, peristomial regions, and coelomic fluid, and no parasites or overt pathological signs were detected. At the end of acclimation, 240 healthy *S. intermedius* (26.49 ± 0.26 g) were randomly selected and distributed into 12 tanks (30 × 50 × 50 cm; 75 L), with 20 individuals per tank and three replicate tanks per treatment group. Daily feeding was conducted at 09:00, with an initial feeding rate of 2% of body weight, adjusted weekly based on growth. Before each feeding, feces and residual feed were removed using a siphon. The remaining feed was collected, dried, and weighed to assess feed intake. The feeding trial lasted for 50 days, and daily supplementation of the water lost in the filtration tank due to the operation of the RAS was carried out to maintain the stability of the water quality and the efficiency of the circulation. The experimental conditions were kept at 17.1 ± 1.2 °C for temperature, 30.2 ± 1.4 ‰ for salinity, and 7.63 ± 0.32 for pH. DO levels remained above 6.65 mg/L.

### 2.3. Biological Sampling and Preservation

Post-trial, the *S. intermedius* was maintained under fasting conditions for 24 h. Subsequently, the number of surviving *S. intermedius* in each tank was recorded, and their individual body weights were measured using a precision balance (±0.01 g). Random selection of six *S. intermedius* from each tank was followed by coelomic fluid collection through the peristomial membrane using sterile syringes. The coelomic fluid was centrifuged at 3000 rpm for 5 min at 4 °C. The resulting supernatant was immediately snap-frozen in liquid nitrogen, transferred into sterile, RNase/DNase-free 2 mL microcentrifuge tubes, and preserved at −80 °C for further analysis of immune and antioxidant parameters.

In addition, ten *S. intermedius* were randomly chosen from each tank for sterile dissection, and the weights of their intestines and gonads were measured. The intestinal and gonadal tissues were transferred to sterile tubes, snap-frozen with liquid nitrogen, and subsequently preserved at −80 °C. Gonad samples were divided, with one portion used for color measurement and the other for proximate and nutritional composition analysis. Portions of the intestinal samples were used to assess digestive enzyme activity and intestinal microbial composition. The remaining samples were used for total RNA extraction, followed by quantitative real-time PCR (qRT-PCR) to analyze the expression of genes related to immunity, inflammation, and antioxidant responses. All assays were conducted in triplicate to enhance the accuracy and reproducibility of the results.

### 2.4. Evaluation of Growth Performance

After the trial, the *S. intermedius* in each tank were evaluated to assess growth performance. Growth performance parameters were computed based on the following equations:

Weight growth rate (WGR, %) = (final weight − initial weight)/initial weight × 100,

Specific growth rate (SGR, %/d) = ln (final weight/initial weight)/breeding days × 100,

Digestive tract index (DTI, %) = final digestive tract weight/final weight × 100,

Gonadosomatic index (GSI, %) = final weight gonad/final body weight × 100,

Feed conversion ratio (FCR, %) = consumed food mass/(final weight − initial weight) × 100,

Survival rate (SR, %) = initial numbers of surviving/final numbers of surviving × 100.

### 2.5. Analysis of Gonad Proximate Composition and Color

The proximate composition of *S. intermedius* gonads (crude protein, lipid, ash, and moisture) was analyzed following the same AOAC methods used for the diet samples.

Gonad color was determined with a ColorCue 2 colorimeter (PANTONE, Carlstadt, NJ, USA), calibrated prior to measurement with a standard white plate (Minolta, Tokyo, Japan). Each gonad sample was measured three times, and the L_0_*, a_0_*, and b_0_* values were recorded. The mean of these values was used as the final color parameter for the sample. The color measurement protocol followed the methodology described by Baião et al. [29] for *S. intermedius* analysis. To investigate the effect of red pepper powder dosage on gonadal color quality, two market-preferred standard colors were used as references, based on the Natural Color System (NCS) chart according to McBride et al. [30]: standard light yellow (L*_s_ = 68.9, a*_s_ = 28.7, b*_s_ = 60.4) and standard light orange-yellow (L*_s_ = 74.6, a*_s_ = 28.7, b*_s_ = 66.1). The total color difference (ΔE) between the gonadal samples and the reference colors was calculated using the CIE 1976 formula:

$$\Delta E_{ab}^{*}=\sqrt{{\left(L_{0}^{*}-L_{s}^{*}\right)}^{2}+{\left(a_{0}^{*}-a_{s}^{*}\right)}^{2}+{\left(b_{0}^{*}-b_{s}^{*}\right)}^{2}}$$

### 2.6. Evaluation of Digestive Enzymes, Immune Capacity, and Antioxidant Responses

Commercial assay kits (Nanjing Jiancheng Bioengineering Institute, Nanjing, China) were utilized for determining intestinal enzyme activities, as well as coelomic immune and antioxidant parameters. Intestinal tissues were homogenized in pre-chilled sterile physiological saline (0.85% NaCl, *w*/*v*) using a tissue homogenizer. The homogenates were centrifuged at 2400× *g* for 10 min at 4 °C, and the resulting supernatants were collected and preserved at 4 °C. Digestive enzyme activities were assayed within 24 h. The concentration of total soluble protein was measured with bovine serum albumin (BSA) as the standard [31].

All parameters were measured using spectrophotometric methods, with specific kit detection wavelengths and catalog numbers listed as follows: Amylase (AMS, 660 nm, C016-1-1), pepsin (PEP, 660 nm, A080-1-1), lipase (LPS, 420 nm, A054-1-1-1), lysozyme (LYS, 530 nm, A050-1-1), alkaline phosphatase (AKP, 520 nm, A059-2), acid phosphatase (ACP, 520 nm, A060-2), catalase (CAT, 405 nm, A007-1-1), superoxide dismutase (SOD, 450 nm, A001-3), glutathione-S-transferase (GST, 412 nm, A004-1-1), glutathione peroxidase (GPX, 412 nm, A005-1), malondialdehyde (MDA, 532 nm, A003-1), and total antioxidant capacity (T-AOC, 425 nm, A015-1-2). All measurements were performed using a UV-Vis spectrophotometer (UV-2700, Shimadzu, Kyoto, Japan) and a microplate reader (Molecular Devices, San Jose, CA, USA), in accordance with the manufacturer’s instructions for each assay kit.

### 2.7. Microbial Community Diversity Assessment

Genomic DNA from *S. intermedius* intestinal contents was extracted using the FastDNA^®^ Spin Kit for Soil (MP Biomedicals, Solon, OH, USA). DNA integrity was confirmed by 1.0% agarose gel electrophoresis, while its concentration was measured using UV–Vis spectrophotometric analysis (Cary 60, Agilent Technologies, Santa Clara, CA, USA). To amplify the V3–V4 variable region of the 16S rRNA gene, the primer pair 338F (5′-ACTCCTACGGGAGGCAGCAG-3′) and 806R (5′-GGACTACHVGGGTWTCTAAT-3′) was employed under standard PCR conditions. Targeted amplification of the V3–V4 region was performed on an ABI GeneAmp^®^ 9700 thermal cycler (Applied Biosystems, Foster City, CA, USA) using the following protocol: 3 min at 95 °C; 29 cycles of 95 °C for 30 s, 53 °C for 30 s, and 72 °C for 45 s; and a final extension of 10 min at 72 °C.

PCR products were recovered from 2% agarose gels and purified using a DNA Gel Extraction Kit (PCR Clean-Up Kit, Yuhua, China). Purified amplicons at equimolar concentrations were pooled and subjected to paired-end sequencing on the Illumina MiSeq PE300 platform (Illumina, San Diego, CA, USA). Sequencing was conducted in line with the standardized procedures of Majorbio Bio-Pharm Technology Co., Ltd. (Shanghai, China). The raw FASTQ files were separate samples and filtered for quality using QIIME. Clean reads were clustered into operational taxonomic units (OTUs, 97% identity) using UPARSE (USEARCH v7.0), and chimeric sequences were filtered out during the process. Taxonomic classification was performed using the SILVA 132 database (16S_bacteria) with a confidence threshold of 0.7.

### 2.8. RNA Extraction and Quantitative Real-Time PCR

RNA extraction and quantitative real-time PCR (qRT-PCR) were conducted following slight adjustments to the method outlined by Zhang et al. [32]. Gene-specific primers were designed by Majorbio (Shanghai, China), and their sequences are provided in Table 2. Total RNA was extracted from *S. intermedius* intestinal tissues using the RNAprep Pure Tissue Kit (DP431, TIANGEN Biotech, Beijing, China). First-strand cDNA synthesis was conducted using the FastKing RT SuperMix Kit (KR118, TIANGEN Biotech, Beijing, China). The concentration and purity of RNA and cDNA were assessed using a UV-Vis spectrophotometer (UV-2700, Shimadzu, Kyoto, Japan). QRT-PCR was performed on a LightCycler^®^ 96 System (Roche, Basel, Switzerland) using SGEXCEL FastSYBR QPCR Premix (SYBR Green I). A melt curve analysis was conducted after each qPCR run to verify amplification specificity and to rule out non-specific products or primer-dimer formation. The 2^−ΔΔCt^ method [33] was employed to assess relative gene expression, using 18S rRNA as the internal reference.

### 2.9. Statistical Analysis

Prior to data processing, normality was tested using the Shapiro–Wilk test, and homogeneity of variance was assessed through Levene’s test. Differences among dietary treatments were evaluated via one-way analysis of variance (ANOVA). When significant effects were observed (*p* < 0.05), Tukey’s Honestly Significant Difference (HSD) test was applied to conduct post hoc comparisons. Data are reported as means ± standard deviations (mean ± SD). All statistical procedures were conducted using SPSS v27.0 (IBM Corp., Armonk, NY, USA).

## 3. Results

### 3.1. Growth Performance and Survival

No mortality was observed in any group of *S. intermedius* throughout the 50-day experimental period. As shown in Table 3, the red pepper powder treatment groups (LL, LM, LH) exhibited significantly higher FBW, WGR, SGR, and GWW than the LC group (*p* < 0.05). Among the treatment groups, the LM group achieved the highest values for all growth parameters, significantly outperforming both the LL and LH groups (*p* < 0.05). Digestive tract weight (DTW) displayed a dose-dependent trend, initially increasing and then declining with higher red pepper powder levels, and peaked in the LM group, showing a significant increase compared the LC group (*p* < 0.05). All treatment groups demonstrated significantly elevated gonadosomatic index (GSI) values compared to the LC group (*p* < 0.05), although no significant variation was found between the treatment groups (*p* > 0.05). Additionally, the feed conversion ratio (FCR) was significantly lower in all treatment groups than in the LC group (*p* < 0.05). The digestive tract index (DTI) remained statistically unchanged across groups (*p* > 0.05).

### 3.2. Gonad Color and Proximate Composition

The proximate composition of *S. intermedius* gonads is presented in Table 4. The crude protein content in the gonads of all treatment groups was significantly higher than that of the LC group (*p* < 0.05). The LM group demonstrated the peak value, although the difference from the LH group was not statistically significant (*p* > 0.05). The crude lipid content in the LM and LH groups was significantly higher than that in the LC group (*p* < 0.05), whereas no significant difference was observed compared to the LL group (*p* > 0.05). In addition, moisture and ash contents of the gonads remained statistically unchanged across groups.

The color attributes of *S. intermedius* gonads are summarized in Table 5. As the dietary inclusion level of red pepper powder increased, both a* and b* values increased significantly compared to the LC group (*p* < 0.05), with the peak values observed in the LH group. No significant difference was detected between the LM and LH groups (*p* > 0.05). The L* value did not exhibit significant variation among groups (*p* > 0.05). In contrast, the color difference parameters ΔE_1_ and ΔE_2_ exhibited a decline as red pepper levels increased. All experimental groups had significantly lower ΔE values than the LC group (*p* < 0.05). ΔE values in the LM and LH groups were also significantly lower than those in the LL group (*p* < 0.05), while no significant difference was detected between LM and LH groups (*p* > 0.05).

### 3.3. Digestive Enzyme Activities

**Figure 2:**
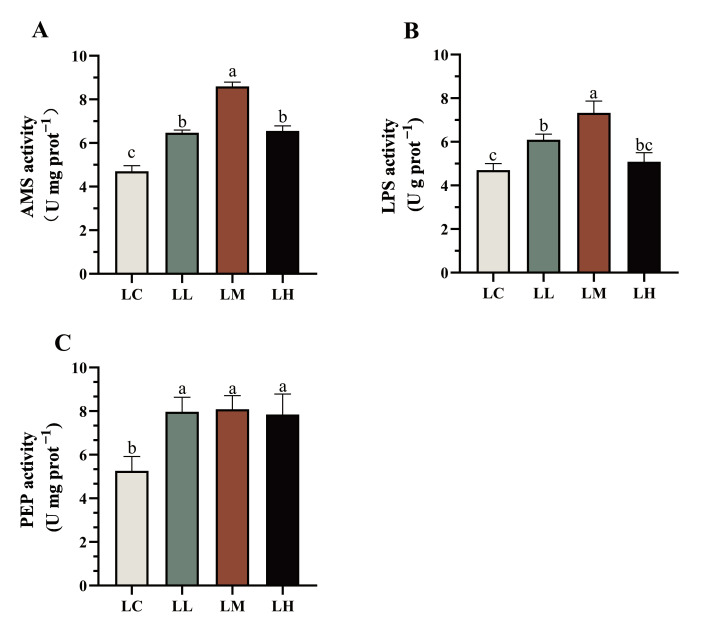
Effects of dietary red pepper powder on digestive enzyme activities in the intestine of *Strongylocentrotus intermedius* (*S. intermedius*). (**A**) amylase (AMS); (**B**) lipase (LPS); (**C**) pepsin (PEP). Values (mean ± SE; *n* = 3) with different letters indicate significant differences (*p* < 0.05).

As shown in Figure 2, AMS and LPS activities in the intestines of *S. intermedius* followed a dose-dependent pattern, initially increasing and then decreasing with higher levels of dietary red pepper powder. AMS activity in the LL, LM, and LH groups was significantly increased relative to the LC group (*p* < 0.05). LPS activity was significantly higher in the LL and LM groups relative to the LC group (*p* < 0.05). Both AMS and LPS activities peaked in the LM group and were significantly elevated relative to the other treatment groups (*p* < 0.05). In addition, PEP activity was significantly increased across treatment groups relative to the LC group (*p* < 0.05), although no statistical differences were detected between the treatment groups (*p* > 0.05).

### 3.4. Immune and Antioxidation Related Parameters

**Figure 3:**
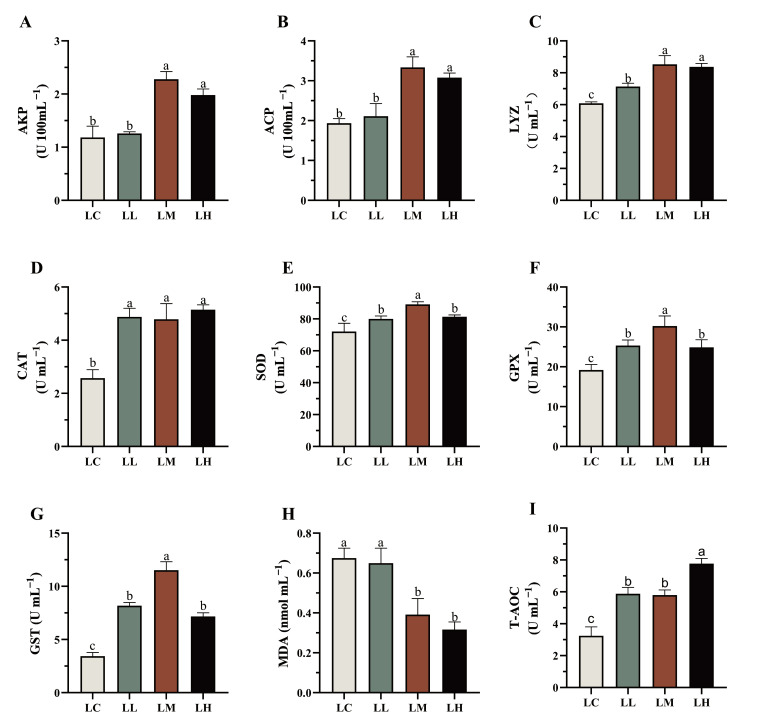
Immune and antioxidant parameters of *S. intermedius* supplemented with different levels of red pepper powder. (**A**) alkaline phosphatase (AKP); (**B**) acid phosphatase (ACP); (**C**) lysozyme (LYZ); (**D**) catalase (CAT); (**E**) superoxide dismutase (SOD); (**F**) glutathione peroxidase (GPX); (**G**) glutathione-S transferase (GST); (**H**) malondialdehyde (MDA); (**I**) total antioxidant capacity (T-AOC); Values (mean ± SE; *n* = 3) with different letters indicate significant differences (*p* < 0.05).

As shown in Figure 3, AKP (A), ACP (B), GPX (F), GST (G), and SOD (E) activities within the coelomic fluid of *S. intermedius* first increased, then decreased as dietary red pepper powder levels rose. AKP and ACP activities were significantly enhanced in the LM and LH groups compared to the LC group (*p* < 0.05), while the LL group did not differ significantly from the LC group (*p* > 0.05).

Activities of GPX, GST, and SOD were significantly increased in all treatment groups relative to the LC group (*p* < 0.05), with the LM group showing significantly higher values than both LL and LH groups (*p* < 0.05). LYZ (C), CAT (D), and T-AOC (I) activities were all significantly elevated in the treatment groups relative to the LC group (*p* < 0.05). LYS activity peaked in the LM and LH groups and was significantly higher than that in the LL group (*p* < 0.05). No significant differences in CAT activity were observed among the treatment groups (*p* > 0.05). T-AOC activity was highest in the LH group, significantly higher than both LL and LM groups (*p* < 0.05). Additionally, MDA (H) levels were significantly reduced in the LM and LH groups relative to the LC group (*p* < 0.05), whereas levels in the LL group did not differ significantly from those in the LC group (*p* > 0.05).

### 3.5. Intestinal Microflora

Alpha diversity parameters were used to assess the richness and evenness of intestinal microbial communities in *S. intermedius* under different dietary treatments. As illustrated in Table 6, the ACE, Chao, and Shannon parameters exhibited an increasing-then-decreasing, peaking in the LM group.

**Figure 4:**
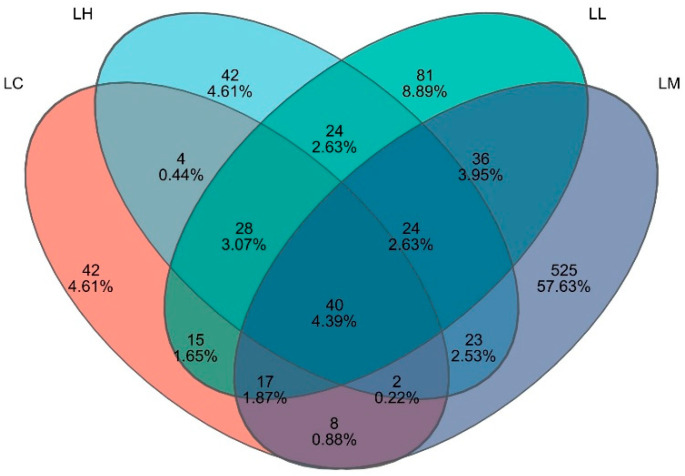
Venn diagram representation of operational taxonomic units (OTUs) in the intestinal microbiota of *S. intermedius* with varying red pepper powder supplementation. LC (control), basal diet; LL, basal diet + 0.5% red pepper powder (*w*/*w*); LM, basal diet + 1% red pepper powder (*w*/*w*); LH, basal diet + 2% red pepper powder (*w*/*w*).

In contrast, the Simpson index followed a decreasing-then-increasing trend, achieving the minimum value in the LM group. According to the Venn diagram (Figure 4), 40 OTUs were shared across all groups, while the LC, LL, LM, and LH groups contained 42, 81, 525, and 42 unique OTUs, respectively. The sequencing coverage was 0.99 across all groups, indicating high data reliability.

**Figure 5:**
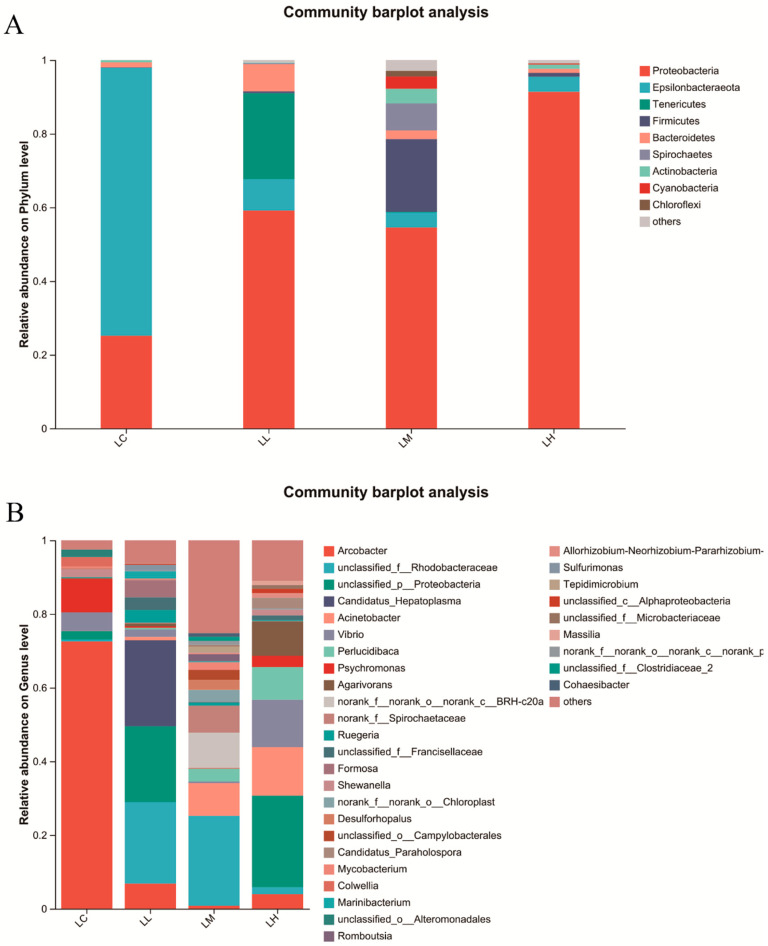
Influence of red pepper powder on the intestinal microbiota composition in *S. intermedius* at phylum (**A**) and genus levels (**B**).

Dietary red pepper powder supplementation markedly influenced the composition of the intestinal microbiota in *S. intermedius* at the phylum (Figure 5A) and genus (Figure 5B) levels. At the phylum level, the predominant bacterial groups included *Proteobacteria*, *Epsilonbacteraeota*, *Tenericutes*, and *Firmicutes*. *Proteobacteria* was the primary phylum, with relative abundances of 25.16%, 59.16%, 54.54%, and 91.4% in the LC, LL, LM, and LH groups, respectively. *Epsilonbacteraeota* showed peak abundance in the LC group (72.73%) and declined in the LL, LM, and LH groups to 8.51%, 4.10%, and 4.06%, respectively. The relative abundance of *Firmicutes* increased in the LM group, reaching its maximum value of 19.77%. At the genus level, the dominant taxa included *Arcobacter*, *unclassified_f__Rhodobacteraceae*, *unclassified_p__Proteobacteria*, and *Candidatus_Hepatoplasma*. Among them, *Arcobacter* showed the greatest relative abundance in the LC group (72.54%) and decreased to 6.81%, 0.81%, and 3.93% in the LL, LM, and LH groups, respectively. The relative abundance of *unclassified_f__Rhodobacteraceae* exhibited an increasing-then-decreasing trend with red pepper powder supplementation, peaking in the LM group at 24%, while its levels were 0.53%, 22.12%, and 1.9% in the LC, LL, and LH groups, respectively.

### 3.6. Physiological Response-Related Genes

**Figure 6:**
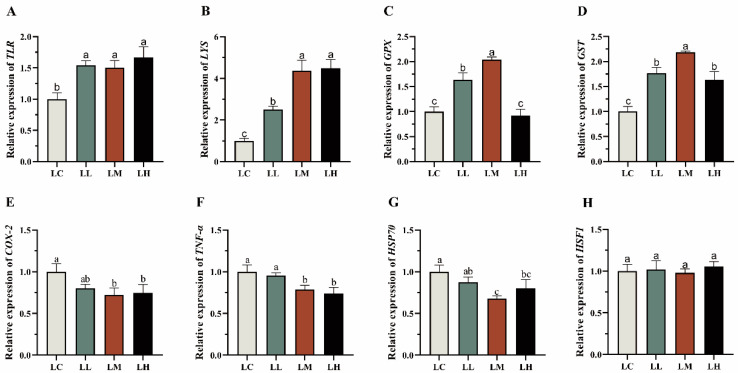
Relative expression of immune, antioxidant, inflammatory, and stress-related genes in *S. intermedius* after 50 days of feeding with different levels of red pepper powder. The LC (control) group transcript levels were normalized to 1, using *18S* as the internal control gene. (**A**) *TLR*: toll-like receptor; (**B**) *LYS*: lysozyme; (**C**) *GPX*: glutathione peroxidase; (**D**) *GST*: glutathione; (**E**) *COX-2*: cyclooxygenase-2; (**F**) *TNF-α*: tumor necrosis factor. (**G**) *HSP70*: heat shock protein 70; (**H**) *HSF1*: heat shock factor protein 1; Values (mean ± SE; *n* = 3) with different letters indicate significant differences (*p* < 0.05).

As shown in Figure 6, the expression of genes associated with immune response, antioxidant defense, inflammatory processes, and stress response was affected by dietary red pepper powder supplementation. With increasing dietary red pepper powder levels, the expression of *TLR* and *LYS* genes in the *S. intermedius* intestine was significantly upregulated relative to the LC group (*p* < 0.05). The expression of *LYS* genes showed an increase correlated with dosage, with peak levels detected in the LM and LH groups, whereas *TLR* expression was generally elevated, no significant differences were observed between the treatment groups (*p* > 0.05). Antioxidant-related genes showed an increasing-then-decreasing expression trend in response to higher red pepper powder levels. Except for the *GPX* gene in the LH group, which showed no significant change (*p* > 0.05), *GPX* and *GST* genes were significantly upregulated in all other treatment groups relative to the LC group (*p* < 0.05). Both genes exhibited peak expression in the LM group, significantly exceeding levels in the LC, LL, and LH groups (*p* < 0.05). Inflammatory markers *COX-2* and *TNF-α* were significantly downregulated in the LM and LH groups compared with the LC group (*p* < 0.05), with no significant difference between the LM and LH groups (*p* > 0.05). Similarly, *HSP70* expression was significantly lower in the LM and LH groups than in the LC group (*p* < 0.05), with the lowest level in the LM group (0.68-fold relative to the LC group), whereas *HSF1* expression showed no significant variation among the groups (*p* > 0.05).

## 4. Discussion

Medicinal plants have been extensively documented to improve a range of physiological functions in aquatic animals, such as growth performance, feeding behavior, digestion, innate immunity, stress tolerance, and antioxidant capacity [35,36,37]. Among these, red pepper (*Capsicum annuum*) is regarded as a safe, eco-friendly, and residue-free natural resource with remarkable anti-inflammatory and antioxidant properties, and has been recognized as an effective functional feed additive [38]. Nevertheless, its potential effects on *Strongylocentrotus intermedius* (*S. intermedius*) remain poorly understood. In this study, dietary supplementation with red pepper powder significantly enhanced the growth performance of *S. intermedius*, strengthened its antioxidant defenses and immune functions, and optimized the composition of the intestinal microbiota. These beneficial effects may be attributed to the enhancement of antioxidant defenses that mitigate oxidative stress, the strengthening of immune responses, and the suppression of inflammatory processes, thereby contributing to the healthy aquaculture and sustainable production of sea urchins. This work provides the first evidence of red pepper powder exerting such effects in echinoderms, thereby broadening its application to sea urchins and emphasizing its promise as a functional feed additive. More critically, the results point to red pepper as a natural antioxidant capable of alleviating physiological stress associated with intensive aquaculture, ultimately contributing to healthier and more resilient sea urchin production.

This study demonstrated that dietary red pepper powder supplementation significantly enhanced the growth performance of *S. intermedius*. The red pepper powder treatment groups (LL, LM, LH) exhibited a significant increase in FBW, WGR, and SGR relative to the control group (LC group), with the medium-dose group (LM) exhibiting the best performance. Similar growth-promoting effects of red pepper powder supplementation have been reported in benni fish (*Mesopotamichthys sharpeyi*), red Mozambique tilapia (*Oreochromis mossambicus*), and rainbow trout (*Oncorhynchus mykiss*) [39,40,41]. In addition, GWW and GSI were elevated in all treatment groups relative to the LC group, with the LM group achieving the utmost gonad quality, suggesting that moderate doses of red pepper powder are more beneficial for gonad development and maturation.

This study further revealed that the LM and LH groups exhibited significantly higher levels of gonadal crude protein and lipid content, along with notable improvements in gonad color characteristics, including increased lightness (L*), redness (a*), and yellowness (b*). Similarly, studies on jewel cichlid (*Hemichromis guttatus*) have reported enhanced pigmentation following dietary red pepper powder supplementation [42]. In addition, the significant reductions in ΔE_1_ and ΔE_2_ values reflect improved gonadal coloration with greater uniformity and chromatic intensity, which may contribute to enhanced visual quality and, consequently, increased market value of the gonads. Such enhancements are likely influenced by bioactive constituents in red pepper, such as carotenoids, which have been implicated in facilitating pigment biosynthesis and deposition in aquatic organisms [43,44]. Furthermore, red pepper powder may contribute to improved growth and gonadal development in sea urchins by enhancing feed conversion efficiency, stimulating digestive enzyme activity, and improving the composition of the intestinal microbiota.

Digestive enzymes are essential regulators of nutrient digestion and absorption efficiency, playing an indispensable role in the ontogenetic progression and physiological development of aquatic species [45]. These enzymes catalyze the breakdown of macromolecules into absorbable units, thus promoting nutrient uptake, energy metabolism, and overall physiological health [46]. In the present study, dietary red pepper powder supplementation enhanced the activity of AMS, LPS, and PEP in *S. intermedius*, with the most pronounced effect observed in the LM group. Increased AMS activity facilitated more efficient hydrolysis and intestinal absorption of carbohydrates, thereby increasing monosaccharide availability [47]. These sugars provide an immediate energy source for aquatic organisms and are critical for sustaining normal metabolic function [48]. Similarly, increased PEP activity enhanced protein digestion and amino acid assimilation [49], potentially contributing to the growth improvements noted in this study. Moreover, higher LPS activity may improve lipid metabolism efficiency and contribute to sustaining energy balance during growth and development [50]. Similar effects have also been reported in fish; for instance, dietary supplementation with garlic powder significantly enhanced digestive enzyme activities in Japanese seabass (*Lateolabrax japonicus*) [51]. This finding is consistent with our observations and further supports the notion that plant-derived additives can modulate digestive physiology, thereby improving nutrient utilization in diverse aquaculture species. Therefore, it is reasonable to infer that appropriate dietary levels of red pepper powder can enhance nutrient absorption in *S. intermedius* by improving digestive efficiency, thereby potentially promoting their growth.

Antioxidant enzyme activity in aquatic animals reflects their physiological adaptability to oxidative stress and serves as an indirect indicator of overall health status [52]. T-AOC is commonly used to evaluate an organism’s overall antioxidant status, as it reflects the combined activity of enzymatic and non-enzymatic antioxidants and is considered a key parameter in assessing resistance to free radical-induced damage [53]. Within the antioxidant defense system, SOD serves as the first line of defense against excess reactive oxygen species (ROS) by catalyzing the dismutation of superoxide anions (O_2_^−^) into hydrogen peroxide (H_2_O_2_), which is then broken down into water and oxygen by CAT, thus sustaining redox balance [54,55]. Furthermore, GPX catalyzes the reduction of peroxides—including hydrogen peroxide and lipid hydroperoxides—by utilizing reduced glutathione, converting peroxides into non-toxic alcohols, together with CAT and SOD, GPX acts synergistically to mitigate oxidative stress and protect cells from oxidative damage [56]. In this study, dietary red pepper powder supplementation significantly enhanced the activity of various antioxidant enzymes in the coelomic fluid of *S. intermedius*, indicating an improved ability to scavenge ROS and heightened resistance to oxidative stress. This effect might stem from the bioactive compounds in red pepper powder, particularly capsaicin. Previous studies have shown that the C7-benzyl carbon and methoxy groups on the phenolic ring of capsaicin confer strong antioxidant and free radical-scavenging properties [57]. Lipid peroxidation generates a variety of byproducts, among which MDA is a commonly used biomarker of oxidative damage, as its levels reflect both the extent of peroxidation and the degree of oxidative injury [58]. In the present study, MDA levels in the coelomic fluid of *S. intermedius* were significantly lower in the LM and LH groups compared with the LC group, suggesting that appropriate dietary supplementation with red pepper powder may attenuate lipid peroxidation and mitigate oxidative damage. This protective effect is likely associated with the coordinated activity of antioxidant enzymes, including SOD, CAT, and GPX, which act synergistically to suppress lipid peroxidation, thereby reducing MDA accumulation and alleviating oxidative stress. [4]. Similar studies have reported that supplementation with natural plant additives such as dragonhead (*Dracocephalum moldavica*) can significantly enhance antioxidant capacity, stress resistance, and health status in zebrafish [9], suggesting that plant-derived compounds may exert conserved antioxidative effects across aquatic species. Therefore, as a natural plant-based functional additive, red pepper powder holds promising potential for improving antioxidant defense, promoting health, and enhancing environmental adaptability in aquatic animals.

As echinoderms (invertebrates), *S. intermedius* lack an adaptive immune system and thus rely entirely on innate immune responses to accommodate environmental changes and potential pathogenic threats [59]. Lysosomal phosphatases are critical to immune regulation and physiological function in aquatic animals [60]. Among these, ACP and AKP are key immune-associated hydrolases responsible for degrading pathogens and damaged cellular components, thereby playing a central role in immune defense [61]. In this study, both ACP and AKP activities in the coelomic fluid of *S. intermedius* were significantly higher in the LM and LH groups, and may thereby support cellular immune functions. LYZ, another critical hydrolytic enzyme in the immune systems of aquatic species, cleaves the β-1,4-glycosidic bonds between N-acetylmuramic acid (NAM) and N-acetylglucosamine (NAG), thereby disrupting bacterial cell walls and inducing cell lysis [62]. Our findings showed that red pepper powder supplementation was associated with increased LYZ activity in the coelomic fluid of *S. intermedius*, suggesting a potential role in enhancing immune defense through the stimulation of immune-related enzymes and possibly improving resistance to environmental pathogens. Similarly, previous studies have demonstrated that dietary supplementation with natural medicinal plant-derived additives can enhance immune enzyme activities and cellular immune responses across diverse aquaculture species. For instance, heartleaf houttuynia (*Houttuynia cordata*) has been shown to stimulate immune performance in sea cucumber (*Apostichopus japonicus*) [35], while gotu kola (*Centella asiatica*) supplementation improved immune parameters in Nile tilapia (*Oreochromis niloticus*) [63]. These findings underscore the broad potential of plant-based additives in strengthening immunity among different aquatic organisms.

An increasing body of evidence indicates that the intestinal microbiota plays a central role not only in nutrient absorption and barrier integrity but also in shaping host immune function and stress resilience. [64,65,66,67]. In the present study, alpha diversity analysis showed that the ACE, Chao, and Shannon indices were highest in the LM group, while the Simpson index was lowest, suggesting that dietary red pepper powder may enhance intestinal microbial diversity and promote the establishment of a more stable microbial community. Such microbial restructuring is often associated with greater resilience to environmental challenges and oxidative stress. These effects may be attributable to bioactive compounds in red pepper, such as polyphenols and capsaicinoids. Capsaicinoid intake has been reported to alter intestinal microbial composition in ways that attenuate inflammation and support metabolic homeostasis [68]. Similarly, plant-derived polyphenols can reshape microbial communities by inhibiting harmful bacteria and enriching beneficial symbionts, thereby exerting a “dual probiotic effect” that favors host health [69]. The phylum-level analysis revealed that *Proteobacteria* was identified as the dominant bacterial group in the *S. intermedius* intestine microbiota, which is consistent with findings reported by Chen et al. [70]. The *Proteobacteria* phylum includes both pathogenic bacteria such as *Arcobacter* and *Vibrio*, which are associated with gastrointestinal inflammation, and beneficial taxa like *Rhodobacteraceae*, which possess complex metabolic networks capable of synthesizing various bioactive compounds. These metabolites support host growth, digestion, and metabolism, and also exhibit antimicrobial properties through the production of secondary metabolites [71,72]. Increasing evidence suggests that *Arcobacteraceae*, a bacterial group within *Campylobacterales*, is emerging as a potential pathogen associated with host inflammatory disorders, particularly those involving the gastrointestinal and reproductive systems [73]. Genus-level analysis indicated that the relative abundance of *Arcobacteraceae* was reduced in the treatment groups relative to the LC group. Although the precise mechanisms remain unclear, these findings suggest that dietary supplementation with red pepper powder may help reduce the abundance of potentially pathogenic bacteria associated with inflammatory processes, thereby indirectly supporting intestinal health through microecological modulation. Concurrently, the observed enrichment of beneficial bacteria like *Unclassified_f__Rhodobacteraceae* in the treatment groups suggests a restructuring of the intestinal microbiota toward a composition more favorable to host health. Members of this family, such as *Rhodobacter sphaeroides*, are capable of synthesizing carotenoids and other bioactive compounds with potent free radical-scavenging and antimicrobial activities. These properties may contribute to strengthening host antioxidant defenses and maintaining cellular redox homeostasis [74]. Collectively, the present findings suggest that dietary supplementation with red pepper powder can modulate the intestinal microbiota in ways that promote host health, potentially enhancing antioxidant capacity through indirect microbiota-mediated pathways. Nevertheless, the specific microbial metabolites and signaling cascades involved remain to be clarified. Future studies integrating metabolomics with functional microbiota manipulation will be essential to elucidate these mechanisms and expand the potential applications of such functional feed additives in aquaculture.

To comprehensively evaluate the regulatory effects of dietary red pepper powder on the physiological status of *S. intermedius*, we analyzed the expression of eight genes—*TLR*, *LYS*, *TNF-α*, *COX-2*, *GPX*, *GST*, *HSP70*, and *HSF1*. These genes are involved in various physiological pathways, including immune recognition, inflammatory signaling, and oxidative stress responses, providing a broad molecular perspective on how *S. intermedius* responds to dietary intervention. Toll-like receptors (TLRs), as crucial pattern recognition receptors in innate immunity, activate the NF-κB signaling pathway and subsequently induce the expression of pro-inflammatory mediators such as tumor necrosis factor-α (*TNF-α*) and cyclooxygenase-2 (*COX-2*), thus playing a central role in the regulation of immune defense and inflammation [75]. The *LYS* gene encodes lysozyme, which contributes to non-specific immunity by hydrolyzing bacterial cell walls and is widely regarded as an important indicator of antimicrobial capacity in the host [76]. Experimental results showed that dietary red pepper powder significantly elevated the expression of immune-related genes (*TLR* and *LYS*) in the intestine of *S. intermedius*. Notably, *LYS* gene expression was significantly elevated in the LM and LH groups, suggesting that moderate to high levels of dietary red pepper powder can enhance non-specific antibacterial defense. This finding is in line with previous studies in *Apostichopus japonicus*, where plant-derived feed additives such as Xiao Chengqi Decoction (containing *Rheum palmatum*, *Magnolia officinalis*, and *Citrus aurantium*) exerted a dose-dependent stimulation of *LYS* gene expression and immune responsiveness, indicating that immune enhancement is closely associated with supplementation levels [77]. Although no dose-dependent differences in *TLR* gene expression were detected among the treatment groups, the consistent enhancement across supplementation levels suggests the presence of a threshold effect, whereby moderate inclusion is sufficient to activate innate immune recognition. This implies that the host may initiate defense mechanisms more rapidly and effectively when challenged by complex environmental stressors such as pathogens, oxidative stress, or high rearing density. A comparable phenomenon of immune priming has also been reported in probiotic studies, where diets supplemented with composite probiotics significantly upregulated both *TLR* and *LYS* transcription in the intestine of *S. intermedius*. These findings indicate that dietary immunomodulators not only strengthen pathogen recognition but also synergistically enhance nonspecific immune responses, thereby reinforcing the overall innate immune defense capacity of the host [78]. Together, these results indicate that dietary red pepper powder may enhance both general immune responsiveness and pathogen recognition capacity, thereby contributing to improved host defense under intensive aquaculture conditions. Additionally, the expression of antioxidant-related genes *GPX* and *GST* peaked in the LM group, exhibiting a dose-dependent pattern consistent with the observed changes in enzymatic activity. Both *GPX* and *GST* are key antioxidant genes involved in detoxifying reactive oxygen species and sustaining cellular redox balance [79], and the parallel trends at transcriptional and enzymatic levels suggest that they may act synergistically to reinforce the antioxidant defense system and improve the organism’s ability to withstand oxidative stress.

TNF-α and COX-2 are well-recognized pro-inflammatory mediators that play central roles in immune regulation and inflammatory processes in aquatic animals [80,81]. For example, in the red-spotted grouper (*Epinephelus akaara*), COX-2 homologs have been shown to modulate inflammatory responses and induce the expression of cytokines, including TNF-α [82]. Similarly, in *S. intermedius*, dietary supplementation with oxidized fish oil significantly upregulated *COX-2* and *TNF-α* genes transcription, highlighting their synergistic involvement under oxidative stress conditions [34]. In contrast, red pepper powder supplementation led to a reduction in *COX-2* and *TNF-α* genes expression in the LM and LH groups, suggesting a potential role in attenuating ROS-mediated inflammatory signaling. Previous studies have emphasized the functional relevance of COX-2 in aquatic species, particularly its role in pathogen-induced inflammation and its regulation through NF-κB and AP-1 signaling pathways [83,84,85]. Within this framework, the observed reduction of these pro-inflammatory mediators in our study suggests that red pepper powder supplementation may interfere with canonical inflammatory cascades. Notably, this anti-inflammatory effect coincided with increased transcription of antioxidant-related genes (*GPX* and *GST*), indicating that red pepper powder may alleviate pro-oxidative processes while strengthening antioxidant defenses to sustain intracellular redox balance.

HSP70 and HSF1 both belong to the heat shock proteins (HSPs) family, which has been extensively documented to play pivotal roles in maintaining protein homeostasis and protecting cells from stress-induced damage, such as oxidative and thermal stress [86]. In the present study, we observed a significant decrease in intestinal *HSP70* gene expression in the LM and LH groups of *S. intermedius*. Given that HSPs are typically induced under stress conditions to mitigate protein damage and sustain cellular homeostasis [87], the downregulation of *HSP70* gene observed here is more likely to reflect a reduction in cellular stress levels under moderate to high supplementation of red pepper powder. Previous in vitro studies have shown that treatment with vitexin (30 μM) reduced intracellular ROS levels and simultaneously downregulated *HSP70* gene expression, providing supporting evidence that decreased *HSP70* gene expression can be regarded as a marker of stress alleviation [88]. HSF1, as a key transcriptional regulator of heat shock proteins, specifically binds to heat shock elements (HSEs) and initiates the transcriptional activation of HSPs [89]. In this study, no significant differences were detected in the transcriptional levels of HSF1 among the experimental groups, which is consistent with the characteristic of *Apostichopus japonicus* to maintain stable transcriptional activity under non-stress conditions [90]. In contrast, *HSP70* gene expression showed significant variation under different treatment conditions. These results indicate that, although the transcription of downstream genes fluctuated, the stable expression pattern of *HSF1* gene suggests that the basal stress-sensing capacity and transcriptional regulatory potential of *S. intermedius* were not disturbed. Overall, moderate supplementation with red pepper powder appeared to enhance the expression of immune- and antioxidant-related genes, while suppressing pro-inflammatory and stress-associated markers. These findings suggest that *S. intermedius* may benefit from improved oxidative stress tolerance, strengthened immune capacity, and greater physiological stability. Such outcomes highlight the potential of red pepper powder as a safe and effective functional feed additive to support health and stress resilience in sea urchin aquaculture under intensive farming conditions.

## 5. Conclusions

In conclusion, dietary supplementation with red pepper powder significantly improved the growth performance of *S. intermedius*, with its beneficial effects primarily linked to enhanced antioxidant defenses, improved immune function, and modulation of intestinal microbiota. At the molecular level, these improvements were associated with the upregulation of antioxidant- and immunity-related genes and the downregulation of stress-response and pro-inflammatory genes, thereby contributing to physiological homeostasis under intensive aquaculture conditions. Furthermore, red pepper powder improved gonadal pigmentation, promoted nutrient deposition, and enhanced gonadal quality. These findings not only highlight red pepper powder as a potent natural antioxidant but also underscore its promise as a green, safe, and sustainable feed additive in sea urchin farming. Importantly, its stress-mitigating capacity carries global significance for sustainable aquaculture. Under the present experimental conditions, ~1% inclusion was identified as the optimal dosage.

## Figures and Tables

**Table 1:** Composition and proximate analysis (dry matter, %) of experimental diets.

Ingredient	Test Diets			
	LC	LL	LM	LH
Fish meal ^1^	40	40	40	40
Soybean meal ^2^	130	130	130	130
Kelp meal ^3^	415	415	415	415
Wheat meal ^4^	300	300	300	300
Wheat gluten ^5^	50	50	50	50
red pepper ^6^	0	5	10	20
Soybean oil	10	10	10	10
Vitamin mixture ^7^	10	10	10	10
Mineral mixture ^8^	10	10	10	10
Monocalcium phosphate	10	10	10	10
Calcium propionate	1.8	1.8	1.8	1.8
Choline chloride	1.2	1.2	1.2	1.2
Microcrystalline cellulose	22	17	12	2
Total	1000	1000	1000	1000
Analyzed nutrients (% on a dry basis)				
Moisture	9.35	9.33	9.37	9.35
Crude lipid	3.05	3.09	3.03	3.06
Crude ash	17.44	17.39	17.40	17.33
Crude protein	22.48	22.45	22.51	20.44
Gross energy (kJ·g^−1^)	16.12	16.13	16.17	16.16

Note: LC (control), basal diet; LL, basal diet + 0.5% red pepper powder (*w*/*w*); LM, basal diet + 1% red pepper powder (*w*/*w*); LH, basal diet + 2% red pepper powder (*w*/*w*). ^1^ Fish meal: the crude protein content is 60% (Technologic De Alimentos S.A., Bogotá, Colombia). ^2^ Soybean: the crude protein content is 40%. ^3^ Kelp meal (Laminaria japonica): 10.31% crude protein. ^4^ Wheat meal: the crude protein content of 13% (China Oil and Foodstuffs Corporation). ^5^ Wheat gluten: 85% crude protein, 2.8% crude lipid, and 0.07% crude fiber. ^6^ Red pepper (*Capsicum annuum*): 9.6% moisture, 13.24% crude protein, 11.16% crude fat, 5.27% ash. ^7^ Vitamin mixture (Guangzhou Nanfang Biotechnology Co., Ltd., Guangzhou, China): 10 g/kg each of vitamin B1, B2, and B6, with a moisture content of less than 10% and anhydrous glucose as the carrier. ^8^ Mineral mixture (Anhui Zhuoke Biotechnology Co., Ltd., Hefei, China): copper (Cu) ranging from 6 to 10 g/kg, iron (Fe) from 30 to 50 g/kg, zinc (Zn) from 6 to 10 g/kg, manganese (Mn) from 15 to 25 g/kg, and magnesium (Mg) from 8 to 13 g/kg.

**Table 2:** Primers for real-time qPCR analysis.

Genes	Primer Name	Primer Sequence (5′−3′)	Efficiency	R^2^	Source or GeneBank
*18s*	*18s*-F	GTTCGAAGGCGATCAGATAC	102.23 ± 4.84	0.997	D14365.1
	*18s*-R	CTGTCAATCCTCACTGTGTC			
*TLR*	*TLR*-F	GAGACGGTACAGGGCTACA	103.2 ± 1.86	0.999	[34]
	*TLR*-R	CGGGCAAAATCCTCACAAG			
*LYZ*	*LYZ*-F	GAGACGGTACAGGGCTACA	105.85 ± 2.84	0.997	[34]
	*LYZ*-R	CGGGCAAAATCCTCACAAG			
*GPX*	*GPX*-F	CGAGTTTGAGAAGCGTGGTG	103.71 ± 2.48	0.996	[34]
	*GPX*-R	GGATCAGCTATGATTGGGTATGG			
*GST*	*GST*-F	CTCGGAGATTCGCTCACCA	97.15 ± 1.75	0.997	[34]
	*GST*-R	GCTGGCTGGAGAAATGAACAA			
*COX-2*	*COX-2*-F	GAGGTGGATAACCGATTGA	105.83 ± 3.26	0.997	MH516324
	*COX-2*-R	GAGGTGGATAACCGATTGA			
*TNF-α*	*TNF-a*-F	GCTGTAACGGCGTTCGTCTCC	95.07 ± 4.46	0.998	MH516331
	*TNF-a*-R	TGGTGTACTTGTGCTGGTTGTTGG			
*HSP70*	*HSP70-F*	ACACTCATCTCGGAGGAG	105.4 ± 5.04	0.99	KF860046.1
	*HSP70-R*	ACACTCATCTCGGAGGAG			
*HSF1*	*HSF1-F*	GGTCTGGAGGATGCGGGAGTAG	105.88 ± 2.87	0.997	591088
	*HSF1-R*	TTGGTGGGTGAATCGCTTGTGAG			

Note: *TLR*: toll-like receptor; *LYZ*: lysozyme; *GPX*: glutathione peroxidase; *GST*: glutathione S-transferase; *COX-2*: cyclooxygenase-2; *TNF-α*: tumor necrosis factor. *HSP70*: heat shock protein 70; *HSP1*: heat shock protein 1.

**Table 3:** Effects of different red pepper powder additive levels growth performance of *Strongylocentrotus intermedius* (*S. intermedius*).

Growth Parameters	LC	LL	LM	LH
IBW (g) ^1^	24.32 ± 0.14 ^a^	23.97 ± 0.2 ^a^	24.27 ± 0.32 ^a^	23.34 ± 0.1 ^a^
FBW (g) ^2^	34.35 ± 0.26 ^c^	35.86 ± 0.27 ^b^	38.12 ± 0.52 ^a^	36.44 ± 0.5 ^b^
WGR (%) ^3^	41.28 ± 1.74 ^c^	49.59 ± 0.22 ^b^	57.1 ± 3.4 ^a^	49.69 ± 2.6 ^b^
SGR (% day^−1^) ^4^	0.69 ± 0.02 ^c^	0.81 ± 0.00 ^b^	0.9 ± 0.04 ^a^	0.81 ± 0.03 ^b^
DTW (g) ^5^	1.44 ± 0.06 ^b^	1.47 ± 0.15 ^ab^	1.61 ± 0.08 ^a^	1.45 ± 0.05 ^b^
DTI (%) ^6^	4.2 ± 0.15 ^a^	4.09 ± 0.06 ^a^	4.22 ± 0.24 ^a^	3.97 ± 0.16 ^a^
GWW (g) ^7^	7.64 ± 0.19 ^c^	8.53 ± 0.12 ^b^	9.3 ± 0.11 ^a^	8.68 ± 0.13 ^b^
GSI (%) ^8^	22.24 ± 0.4 ^b^	23.8 ± 0.24 ^a^	24.39 ± 0.41 ^a^	23.82 ± 0.34 ^a^
FCR ^9^	2.05 ± 0.07 ^a^	1.81 ± 0.01 ^b^	1.69 ± 0.08 ^b^	1.82 ± 0.08 ^b^
SR (%) ^10^	100 ± 0.00 ^a^	100 ± 0.00 ^a^	100 ± 0.00 ^a^	100 ± 0.00 ^a^

Values (mean ± SE; *n* = 3) with different superscripts differ significantly (*p* < 0.05). ^1^ IBW, initial body weight. ^2^ FBW, final body weight. ^3^ WGR, weight gain rate. ^4^ SGR, specific growth rate. ^5^ DTW, digestive tract weight. ^6^ DTI, digestive tract index. ^7^ GWW, gonad wet weight. ^8^ GSI, gonadosomatic index. ^9^ FCR, feed conversion ratio. ^10^ SR, survival rate.

**Table 4:** Effect of different red pepper powder additive levels on gonad proximate composition of *S. intermedius* (dry matter, %).

Parameter	Test Diets			
	LC	LL	LM	LH
Moisture (%)	78.09 ± 0.78 ^a^	77.37 ± 0.14 ^a^	77.14 ± 0.17 ^a^	77.09 ± 0.26 ^a^
Crude protein (%)	10.49 ± 0.12 ^c^	11.19 ± 0.18 ^b^	11.78 ± 0.13 ^a^	11.76 ± 0.1 ^a^
Crude lipid (%)	7.84 ± 0.2 ^b^	8.17 ± 0.23 ^ab^	8.53 ± 0.14 ^a^	8.57 ± 0.06 ^a^
Crude ash (%)	2.35 ± 0.02 ^a^	2.33 ± 0.06 ^a^	2.36 ± 0.04 ^a^	2.33 ± 0.03 ^a^

Values (mean ± SE; *n* = 3) with different superscripts differ significantly (*p* < 0.05).

**Table 5:** Effect of different red pepper powder additive levels on gonad color of *S. intermedius*.

Parameter	Test Diets			
	LC	LL	LM	LH
L* ^1^	67.53 ± 0.17 ^a^	66.96 ± 0.73 ^a^	66.85 ± 0.49 ^a^	67.03 ± 0.49 ^a^
a* ^2^	13.19 ± 0.31 ^c^	16.07 ± 0.32 ^b^	17.3 ± 0.58 ^ab^	18.36 ± 0.94 ^a^
b* ^3^	30.22 ± 0.69 ^c^	32.45 ± 0.21 ^b^	35.83 ± 0.6 ^a^	36.3 ± 0.44 ^a^
ΔE_1_^4^	33.96 ± 0.73 ^a^	30.73 ± 0.21 ^b^	27.18 ± 0.32 ^c^	26.31 ± 0.45 ^c^
ΔE_2_ ^5^	39.72 ± 0.73 ^a^	36.75 ± 0.13 ^b^	33.27 ± 0.42 ^c^	32.45 ± 0.36 ^c^

Values (mean ± SE; *n* = 3) with different superscripts differ significantly (*p* < 0.05). ^1^ L*: Represents the lightness of the color. ^2^ a*: Indicates the degree of redness. ^3^ b*: Indicates the degree of yellowness. ^4^ ΔE_1_: The color difference from the light orange yellow standard. ^5^ ΔE_2_: The color difference from the light yellow standard. A smaller ΔE value suggests a closer alignment with the reference color.

**Table 6:** Effect of different red pepper powder additive levels on alpha diversity indices of *S. intermedius*.

Parameter	Test Diets			
	LC	LL	LM	LH
Shannon	1.43	2.95	4.11	3.13
Chao1	171.5	283	698.85	188.36
Ace	180.99	289.98	696.27	189.2
Simpson	0.53	0.12	0.05	0.1
Coverage	0.99	0.99	0.99	0.99

Note: values are based on a mixed sample from three *S. intermedius* per treatment group.

## Data Availability

All relevant data are presented within the paper.
